# *β*-Hydroxybutyrate induces microglial M2 polarization by inhibiting the NF-κB/NLRP3 pathway to ameliorate cerebral ischemia–reperfusion injury

**DOI:** 10.3389/fneur.2026.1847958

**Published:** 2026-08-12

**Authors:** Qiong Chen, Shui Cheng, Chang Huang, Ruping Xiang

**Affiliations:** 1 Department of Neurology, Ward 3, The Fourth Hospital of Changsha, Binshui Xincheng Campus, Changsha, Hunan, China; 2 Department of Neurology, Ward 2, The Fourth Hospital of Changsha, Yuelu Campus, Changsha, Hunan, China; 3 Department of Neurology, Ward 1, The Fourth Hospital of Changsha, Yuelu Campus, Changsha, Hunan, China

**Keywords:** cerebral ischemia–reperfusion injury, ketone bodies, neuroprotection, NF-κB/NLRP3 pathway, obesity

## Abstract

**Objective:**

To investigate the neuroprotective effects of *β*-hydroxybutyrate (BHB) on cerebral ischemia–reperfusion injury (CIRI) and its underlying molecular mechanisms; to clarify the association between ketone bodies and the severity of CIRI under obese conditions.

**Methods:**

CIRI rat model was established using modified middle cerebral artery occlusion/reperfusion (MCAO/R), while obesity was induced using high fat diet. BHB was administered via subcutaneous osmotic pumps. Lipopolysaccharide (LPS) was intraperitoneally injected to forcibly activate the nuclear factor-κB (NF-κB)/NOD-like receptor protein 3 (NLRP3) pathway. Neurological deficits were evaluated using the Zea–Longa 5-point scoring system. Serum BHB levels were measured by colorimetric assay. Immunofluorescence was performed to detect CD86 (M1 phenotype) and CD206 (M2 phenotype) levels. Inflammatory cytokines in brain tissues were measured. Levels of phosphorylated p65 (p-p65), cleaved caspase-1, and ionized calcium-binding adapter molecule 1 (Iba1) were analyzed by Western blotting.

**Results:**

CIRI rats with obesity exhibited increased serum BHB levels, reduced neurological deficit scores, and markedly decreased infarct volumes. Moreover, levels of p-p65, cleaved caspase-1, Iba1, and pro-inflammatory cytokines, were decreased, while Iba1^+^CD206^+^ co-localization was increased. BHB treatment for CIRI rats with normal weight reduced neurological deficit scores, increased serum BHB, and decreased infarct volume. Following further LPS administration, neurological deficit scores and infarct volume were enhanced, with no difference in serum BHB levels.

**Conclusion:**

Elevated endogenous BHB under obese conditions exerts neuroprotective effects against CIRI, and exogenous BHB can mimic this effect. Mechanistically, BHB inhibits activation of the NF-κB/NLRP3 pathway and promotes microglial polarization toward the anti-inflammatory M2 phenotype, thereby alleviating CIRI.

## 1 Introduction

Cerebral ischemia–reperfusion injury (CIRI) is a common secondary complication following thrombolysis and endovascular thrombectomy for acute ischemic stroke. Neuroinflammation plays a central role in CIRI progression, characterized by excessive activation of inflammatory signaling, overproduction of pro-inflammatory cytokines, and dysregulated microglial polarization, ultimately leading to aggravated neuronal injury and poor functional recovery (1–3). Obesity, a well-established risk factor for cerebrovascular disease, has traditionally been associated with worse stroke outcomes due to chronic low-grade inflammation and metabolic dysfunction (4). However, obesity is also accompanied by altered metabolic states, including elevated levels of endogenous ketone bodies (5), whose potential impact on CIRI remains insufficiently explored.

Among these metabolic changes, ketone bodies, particularly *β*-hydroxybutyrate (BHB), have attracted increasing attention. BHB, a major endogenous ketone body elevated under obese conditions, has been reported to exert anti-inflammatory and antioxidative effects (6, 7). However, whether obesity-associated increases in endogenous BHB confer neuroprotection against CIRI remains unclear. Additionally, the nuclear factor-κB (NF-κB)/NOD-like receptor protein 3 (NLRP3) inflammasome pathway is central to the regulation of neuroinflammation. Its activation after CIRI promotes caspase-1 cleavage, leading to the maturation and release of pro-inflammatory cytokines such as interleukin-1β (IL-1β) and interleukin-18 (IL-18), while driving microglial polarization toward the pro-inflammatory M1 phenotype (8, 9). Notably, BHB has been identified as a potential endogenous inhibitor of the NLRP3 inflammasome (10); however, its role in modulating the NF-κB/NLRP3 pathway in the context of obesity-associated CIRI has not been fully elucidated.

Therefore, this study focuses on the obesity–CIRI–BHB–NF-κB/NLRP3 axis. By establishing an obesity-associated CIRI rat model and integrating exogenous BHB intervention with pharmacological activation of the NF-κB/NLRP3 pathway, we systematically investigated the neuroprotective effects and molecular mechanisms of BHB *in vivo*. The novelty of this study lies in revealing that elevated endogenous BHB under obese conditions serves as a compensatory protective factor in CIRI, and in demonstrating that BHB exerts neuroprotection by suppressing NF-κB/NLRP3-mediated neuroinflammation and promoting microglial M2 polarization. These findings may provide new insights into the dual role of obesity in stroke and identify BHB as a potential endogenous therapeutic target for CIRI.

## 2 Materials and methods

### 2.1 Materials

#### 2.1.1 Experimental animals

Ninety male Sprague–Dawley (SD) rats (6–8 weeks old) were housed under standard laboratory conditions (22 ± 2 °C, 12 h light/dark cycle) with free access to food and water. All procedures were approved by the Institutional Animal Care and Use Committee of The Fourth Hospital of Changsha (Approval Number: CSSDSYY-YXLL-SC-2024-02-30).

#### 2.1.2 Reagents

BHB (purity ≥98%; 300–85-6; Must Biotechnology, Chengdu, China); lipopolysaccharide (LPS; purity ≥99%; L2880; Sigma-Aldrich, St Louis, MO, USA); dimethyl sulfoxide (DMSO; D2650; Sigma-Aldrich); 4% paraformaldehyde (G1101; Servicebio, Wuhan, China); 0.9% sodium chloride injection (Hengrui Pharmaceuticals, Jiangsu, China); high-fat diet (HFD; TP23300; Trophic Animal Feed, Nantong, China); standard diet (TP2018; Trophic Animal Feed); middle cerebral artery occlusion/reperfusion (MCAO/R) monofilaments (2838-50/A4; Cinontech, Beijing, China); radioimmunoprecipitation assay (RIPA) lysis buffer (P0013B; Beyotime, Shanghai, China); bicinchoninic acid (BCA) protein assay kit (BCA02; Dingguo Changsheng, Beijing, China); enhanced chemiluminescence (ECL) reagent (MA0186; Meilunbio, Dalian, China); primary antibodies against phosphorylated p65 (p-p65; 3,033; Cell Signaling Technology, Danvers, MA, USA), p65 (80979-1-RR; Proteintech, Rosemont, IL, USA), cleaved caspase-1 (AF4005; Affinity, Milwaukee, WI, USA), ionized calcium-binding adapter molecule 1 (Iba1; PA5-27436; Thermo Fisher Scientific, Waltham, MA, USA), and glyceraldehyde-3-phosphate dehydrogenase (GAPDH; A19056; ABclonal, Woburn, MA, USA); horseradish peroxidase (HRP)-conjugated goat anti-rabbit IgG (GB23303; Servicebio) and goat anti-mouse IgG (GB23301; Servicebio); IL-1β enzyme-linked immunosorbent assay (ELISA) kit (E-EL-R0012; Elabscience, Wuhan, China); IL-18 ELISA kit (E-EL-R0567; Elabscience); primary antibodies for immunofluorescence: Iba1 (GB153502-100; Servicebio), CD86 (GB150054-100; Servicebio), CD206 (GB113497-100; Servicebio); Alexa Fluor 488-conjugated goat anti-rabbit secondary antibody (GB25303; Servicebio); Alexa Fluor 594-conjugated goat anti-rabbit secondary antibody (GB25301; Servicebio); 4′,6-diamidino-2-phenylindole (DAPI) staining solution (G1012; Servicebio); BHB colorimetric assay kit (S0190; Beyotime). Nigericin (S2863, Selleck, USA).

#### 2.1.3 Instruments

Electrophoresis power supply (WIX-EP600, WIX Technology, Beijing, China); electrophoresis tank (WIX-miniPRO4, WIX Technology); transfer system (WIX-miniBLOT4, WIX Technology); chemiluminescence imaging system (SH-523, Shenhua Bio, Hangzhou, China); orbital shaker (SLA-O3000-S, SCILOGEX, Rocky Hill, CT, USA); paraffin microtome (RM2255, Leica Microsystems, Germany); inverted fluorescence microscope (TE2000-S, Nikon, Japan); laser scanning confocal microscope (LSM880, Zeiss, Germany); upright microscope (BX53, Olympus, Japan); microplate reader (FC, Thermo Fisher Scientific).

### 2.2 Methods

#### 2.2.1 Rat grouping and treatment

After 1 week of acclimatization, rats were divided into the NM group (*n* = 70; standard diet) and the OB group (*n* = 20; receiving HFD for 6 weeks: 60% fat, 20% carbohydrate). Sample size was calculated in advance based on preliminary experimental data to guarantee statistical reliability. The unequal initial sample size between normal and obese groups was preset because high-fat diet-induced obesity modeling has a higher failure rate. The criterion for determining obesity is that the body weight is at least 20% higher than the average value of the normal control group, which is used to verify whether the obesity model is successfully established. Eighteen obese rats were successfully modeled and randomly assigned to OB-Sham and OB-CIRI groups (*n* = 9 each). After MCAO/R surgery, 1 rat in the OB-CIRI group was excluded owing to perioperative death or unsuccessful model formation; no rats were eliminated in the OB-Sham group. The final sample size of both OB subgroups was Final n = 8 for subsequent testing.

The NM cohort was randomly divided into seven subgroups with 10 rats assigned to each group: NM-Sham, NM-CIRI, NM-CIRI + normal saline, NM-CIRI + BHB, NM-CIRI + BHB + DMSO, NM-CIRI + BHB + LPS, and NM-CIRI + BHB + Nigericin. All subgroups except NM-Sham underwent MCAO/R cerebral ischemia–reperfusion surgery. Two rats were excluded from each surgical subgroup due to intraoperative hemorrhage, postoperative death or invalid neurological scoring; no animals were excluded from the NM-Sham sham-operated group. Consistently, all seven NM subgroups reached a unified final sample size of Final n = 8 for all subsequent biochemical, immunofluorescence, TTC staining and Western blot analyses. Detailed enrollment and exclusion information is presented in the CONSORT-based animal flow chart (Supplementary Figure S1).

For treatment, BHB was delivered via subcutaneous osmotic pumps (1 μL/h; 1.6 mmol/kg/24 h) implanted 5 min before reperfusion. LPS (1 mg/kg) was intraperitoneally injected 30 min before reperfusion. Normal saline was continuously infused subcutaneously at 1 μL/h for 4 h via osmotic pumps as pump vehicle control; equal-volume DMSO was intraperitoneally injected at 30 min prior to reperfusion as solvent control matching LPS administration. Nigericin (5 mg/kg), dissolved in an equal volume of DMSO, was intraperitoneally administered 30 min prior to reperfusion to specifically trigger NLRP3 inflammasome assembly. The Nigericin group was set as a parallel verification group for LPS-induced pathway activation.

#### 2.2.2 MCAO/R model establishment

The MCAO/R model was established using a modified Zea–Longa method. Rats were anesthetized via intraperitoneal injection (i.p.) with 3% pentobarbital sodium at a dosage of 45 mg/kg body weight. Supplementary low-dose anesthetic (1/4 of initial dosage) was administrated intraperitoneally during surgery if animals showed limb withdrawal reflex to pain stimulation. A monofilament was inserted into the internal carotid artery to a depth of 18–20 mm to occlude the cerebral artery. After 1 h of ischemia, the filament was withdrawn to allow reperfusion, the surgical field was washed with preheated sterile normal saline. Layered suture was conducted for separated neck muscle and skin tissue. Antibiotic ointment was topically applied on incision sites to reduce infection risk. Postoperatively, all rats were transferred into temperature-controlled incubators at 37 °C for postoperative resuscitation from anesthesia. After full consciousness recovery, rats were raised routinely with free food and water supply and received daily postoperative care and health monitoring in accordance with approved animal ethical protocols.

Neurological deficits were assessed using the Zea–Longa scoring system (11): 0, no deficit; 1, failure to fully extend contralateral forelimb; 2, circling; 3, falling to the contralateral side; 4, no spontaneous walking with loss of consciousness. Rats scoring 1–3 were considered successful models.

Finally, 8 rats per group (OB-CIRI, NM-CIRI, NM-CIRI + BHB, NM-CIRI + saline, NM-CIRI + BHB + LPS, NM-CIRI + BHB + DMSO and NM-CIRI + BHB + Nigericin) were included for subsequent analyses, with mortality rates ranging from 10 to 20%.

#### 2.2.3 Blood and tissue sampling

All blood and tissue sampling procedures were performed strictly at three predefined time points, including 6 h, 24 h, and 72 h after MCAO/R reperfusion. Prior to sampling, rats were deeply anesthetized via intraperitoneal injection with 3% pentobarbital sodium (45 mg/kg) at the corresponding post-reperfusion time points. After the disappearance of pain reflexes and complete anesthesia confirmation, abdominal aortic blood was rapidly collected using sterile syringes. The harvested whole blood was placed in anticoagulant-free sterile EP tubes, allowed to stand still at room temperature for 30 min, and then centrifuged at 3500 rpm for 15 min at 4 °C. The supernatant serum was aliquoted immediately and stored at −80 °C for subsequent detection of serum BHB levels.

Following complete blood collection, all experimental rats were sacrificed by rapid decapitation. The whole brain tissues were quickly isolated on an ice-cold sterile platform, rinsed with pre-cooled phosphate-buffered saline to remove residual blood, and dried gently. The brain samples were divided and preserved according to subsequent experimental requirements: partial tissues were fixed with 4% paraformaldehyde for immunofluorescence staining, and the remaining tissues were frozen and stored at −80 °C for Western blot and qPCR molecular detection. All sampling operations were completed under sterile and low-temperature conditions to ensure the stability of biological samples.

#### 2.2.4 Measurement of serum BHB

Use the BHB colorimetric assay kit in accordance with the manufacturer’s instructions. Standards and serum samples were added to a 96-well plate, followed by sequential addition of enzyme working solution and chromogenic reagent, with incubation in the dark, and then the stop solution. Absorbance was measured at 550 nm using a microplate reader. Serum BHB concentrations were calculated based on the standard curve, and all samples were assayed in duplicate to ensure accuracy.

#### 2.2.5 2,3,5-Triphenyltetrazolium chloride (TTC) staining

At 72 h after model establishment, rats were deeply anesthetized and decapitated. Brains were rapidly removed, frozen at −20 °C for 15 min, and cut into five coronal sections (approximately 2 mm thick). Sections were incubated in 2% TTC solution at 37 °C for 20 min in the dark, then fixed in 4% paraformaldehyde. After removing excess fixative, sections were placed on a black background. Normal tissue appeared red, whereas infarcted areas were white. Images were captured, and infarct areas were quantified using Image-Pro Plus 6.0. Infarct volume was calculated as total infarct area × slice thickness (2 mm), and the percentage of infarct volume was recorded (12).

#### 2.2.6 Immunofluorescence

Brain tissues were collected 72 h after modeling, fixed by perfusion with 4% paraformaldehyde, dehydrated in graded sucrose, and sectioned into 10–15 μm frozen coronal slices, which were stored at −20 °C. Sections underwent antigen retrieval using citrate buffer, followed by blocking with 0.3% Triton X-100 and 5% goat serum at room temperature for 1 h. Primary antibodies were applied and incubated overnight at 4 °C. The next day, sections were rewarmed for 30 min, washed with phosphate-buffered saline (PBS), and incubated with fluorescent secondary antibodies (1:400) for 1 h at room temperature in the dark. Nuclei were stained with DAPI for 10 min, and sections were mounted with anti-fade medium. Images from the ischemic region and five surrounding fields were captured under identical settings using a laser scanning confocal microscope. ImageJ software was used to quantify positive cells, fluorescence intensity, and the ratios of Iba1^+^CD86^+^ and Iba1^+^CD206^+^ cells. All procedures were performed under light-protected conditions with strict control of incubation and washing steps (13).

#### 2.2.7 ELISA

Brain tissues were homogenized (10%) and centrifuged (3,000 rpm, 10 min). Supernatants were collected, and IL-1β and IL-18 levels were measured using ELISA kits (14).

#### 2.2.8 Western blot assay

Rats were perfused with 0.9% sodium chloride (NaCl), and hippocampal tissues from the ischemic region were rapidly isolated on ice. Tissues were homogenized in RIPA lysis buffer (20 × volume) with two grinding beads at 60 Hz for 60 s (twice). Lysates were incubated on ice for 30 min with vortexing every 10 min, followed by centrifugation at 4 °C and 12,000 rpm for 15 min. Supernatants were collected, diluted 10-fold, and protein concentrations were determined using the bicinchoninic acid (BCA) assay. Samples were boiled at 100 °C for 5 min for denaturation and stored at −80 °C. Proteins were separated by SDS-PAGE at 80 V, transferred to membranes at 400 mA for 40 min, blocked with 5% non-fat milk for 1 h, and incubated with primary and secondary antibodies before detection.

### 2.3 Statistical analysis

Data were analyzed using SPSS 23.0. Results are presented as mean ± standard deviation (x̄ ± s). Prior to analysis, the Shapiro–Wilk test was used to assess normality and Levene’s test was used to evaluate homogeneity of variance. For data satisfying normal distribution and homogeneity of variance, one-way analysis of variance (ANOVA) was performed, followed by the least significant difference (LSD) post-hoc test for pairwise comparisons. When the assumption of homogeneity of variance was not met, Tamhane’s T2 test was applied. Nonparametric tests were used for data that did not conform to normal distribution. All analyses were two-tailed, and a *p* value < 0.05 was considered statistically significant.

All investigators responsible for Zea–Longa neurological scoring, TTC-based infarct volume quantification and immunofluorescence image analysis were kept blinded to animal group allocation throughout data collection and quantification to eliminate potential observer bias.

## 3 Results

### 3.1 Verification of metabolic and inflammatory characteristics in obesity models

**Figure 1:**
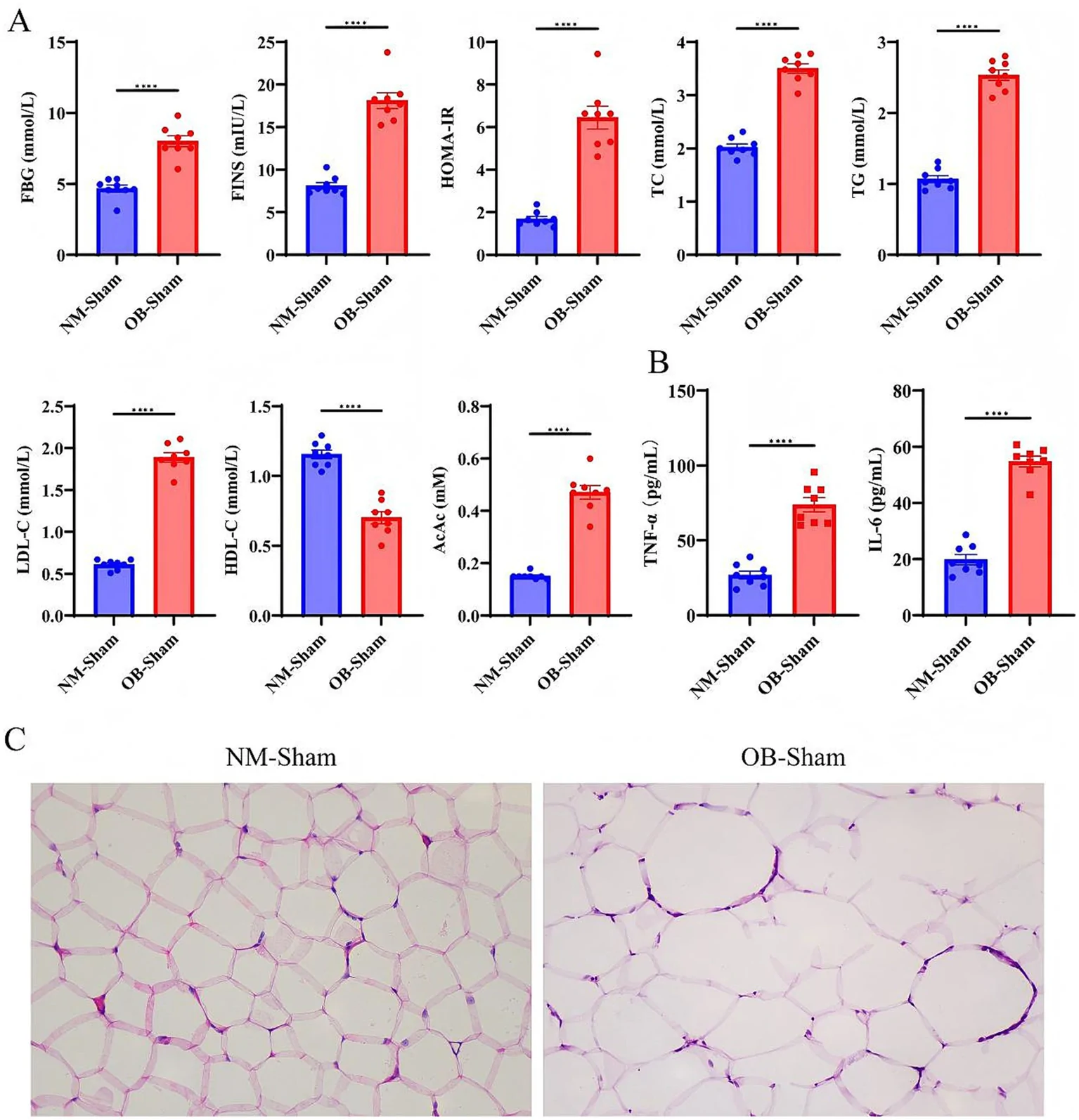
Verification of metabolic, inflammatory and histopathological characteristics of obese model rats. **(A)** Detection of serum FBG, FINS, HOMA-IR, TC, TG, LDL-C, HDL-C and AcAc levels. **(B)** Serum TNF-*α* and IL-6 levels detected by ELISA. **(C)** Histopathological observation of epididymal adipose tissue. *n* = 8 each group. *p < 0.05, **p < 0.01, ***p < 0.001, ****p < 0.0001. NM-Sham: normal sham group; OB-Sham: obese sham group.

Serum metabolic analysis demonstrated that compared with the NM-Sham group, the levels of fasting blood glucose (FBG), fasting insulin (FINS), homeostatic model assessment for insulin resistance (HOMA-IR), total cholesterol (TC), triglycerides (TG) and low-density lipoprotein cholesterol (LDL-C) were significantly increased in the OB-Sham group (*p* < 0.0001). Meanwhile, high-density lipoprotein cholesterol (HDL-C) was significantly decreased, whereas the ketone body marker acetoacetic acid (AcAc) was markedly upregulated in the OB-Sham group (*p* < 0.0001) (Figure 1A). ELISA results showed that the serum levels of pro-inflammatory factors TNF-*α* and IL-6 were significantly higher in the OB-Sham group than those in the NM-Sham group (*p* < 0.0001) (Figure 1B). Histopathological observation of epididymal adipose tissues showed that adipocytes in the NM-Sham group had regular shape and uniform size, with no apparent inflammatory infiltration. In comparison, adipocytes in the OB-Sham group exhibited obvious hypertrophy and heterogeneous proliferation, along with extensive macrophage infiltration (Figure 1C).

### 3.2 Obesity elevates endogenous ketone levels and alleviates neurological injury after CIRI

**Figure 2:**
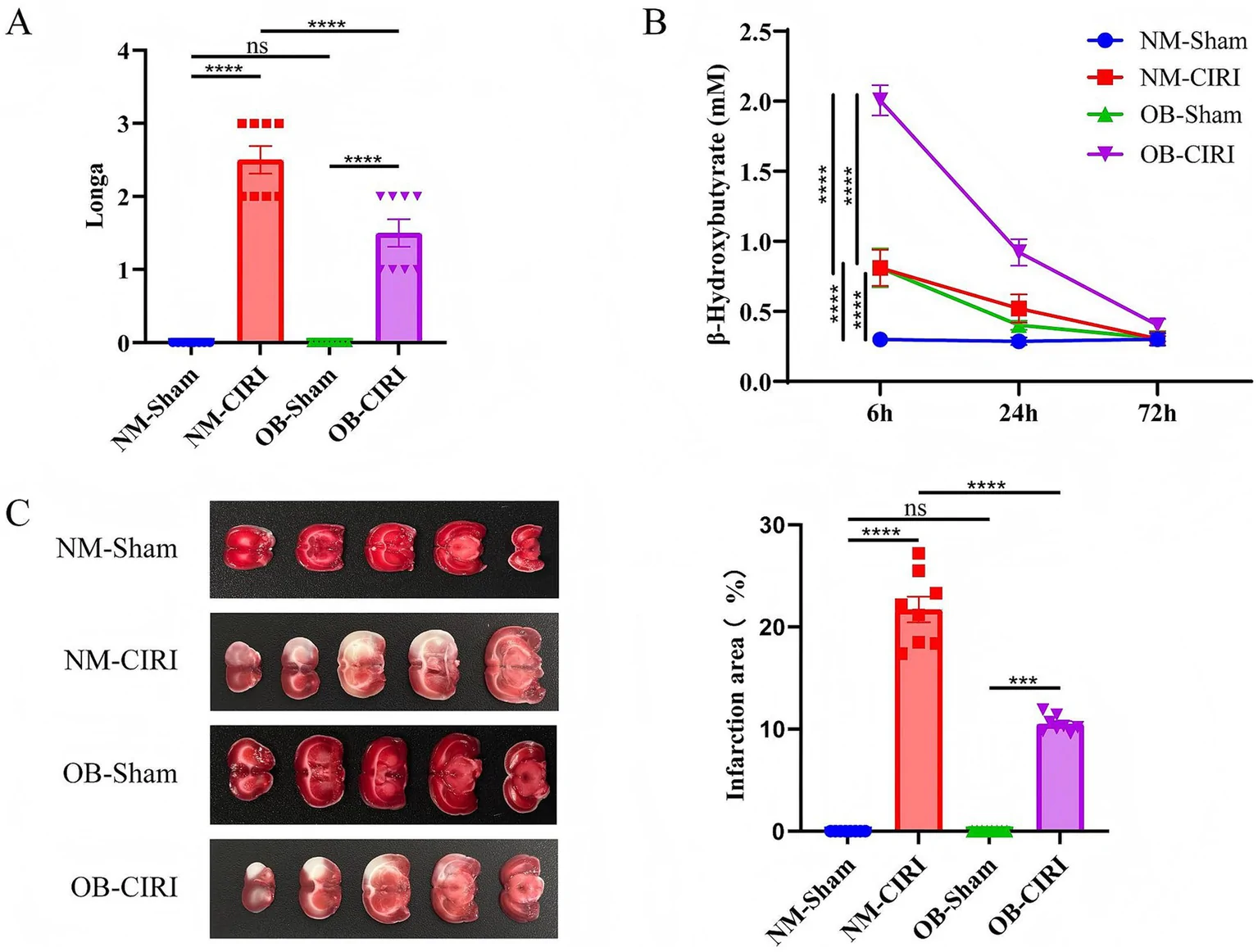
Obesity increases endogenous ketone levels and attenuates neurological injury after CIRI. **(A)** Zea–Longa neurological deficit scores at 72 h after CIRI. **(B)** Serum BHB levels at 6 h, 24 h, and 72 h after CIRI. **(C)** TTC staining of brain tissue and quantitative analysis of infarct volume percentage at 72 h after CIRI. n = 8 each group. *p < 0.05, **p < 0.01, ***p < 0.001, ****p < 0.0001. NM-Sham, normal sham group; OB-Sham, obese sham group; NM-CIRI, normal CIRI group; OB-CIRI, obese CIRI group.

Compared with the NM-Sham group, Zea–Longa scores were significantly increased in the NM-CIRI group (*p* < 0.0001). Similarly, compared with the OB-Sham group, scores were significantly increased in the OB-CIRI group (*p* < 0.0001). Notably, Zea–Longa scores were significantly lower in the OB-CIRI group than in the NM-CIRI group (*p* < 0.05) (Figure 2A). Serum BHB levels were significantly higher in the OB-Sham group than in the NM-Sham group at 6 h and 24 h, both NM-CIRI and OB-CIRI groups exhibited further elevated serum BHB (all *p* < 0.0001), with the OB-CIRI group showing the highest BHB concentration (*p* < 0.0001) (Figure 2B). Compared with the NM-CIRI group, serum BHB levels in the OB-CIRI group were significantly higher at 6 h and 24 h (*p* < 0.05), with no significant difference at 72 h. Infarct volumes in both NM-CIRI and OB-CIRI groups were significantly increased compared with their respective sham groups (*p* < 0.0001) (Figure 2C). No significant difference was observed between NM-Sham and OB-Sham groups. However, infarct volume was significantly reduced in the OB-CIRI group compared with the NM-CIRI group (*p* < 0.05).

### 3.3 Obesity-associated neuroprotection is linked to inhibition of the NF-κB/NLRP3 pathway and induction of microglial M2 polarization

**Figure 3:**
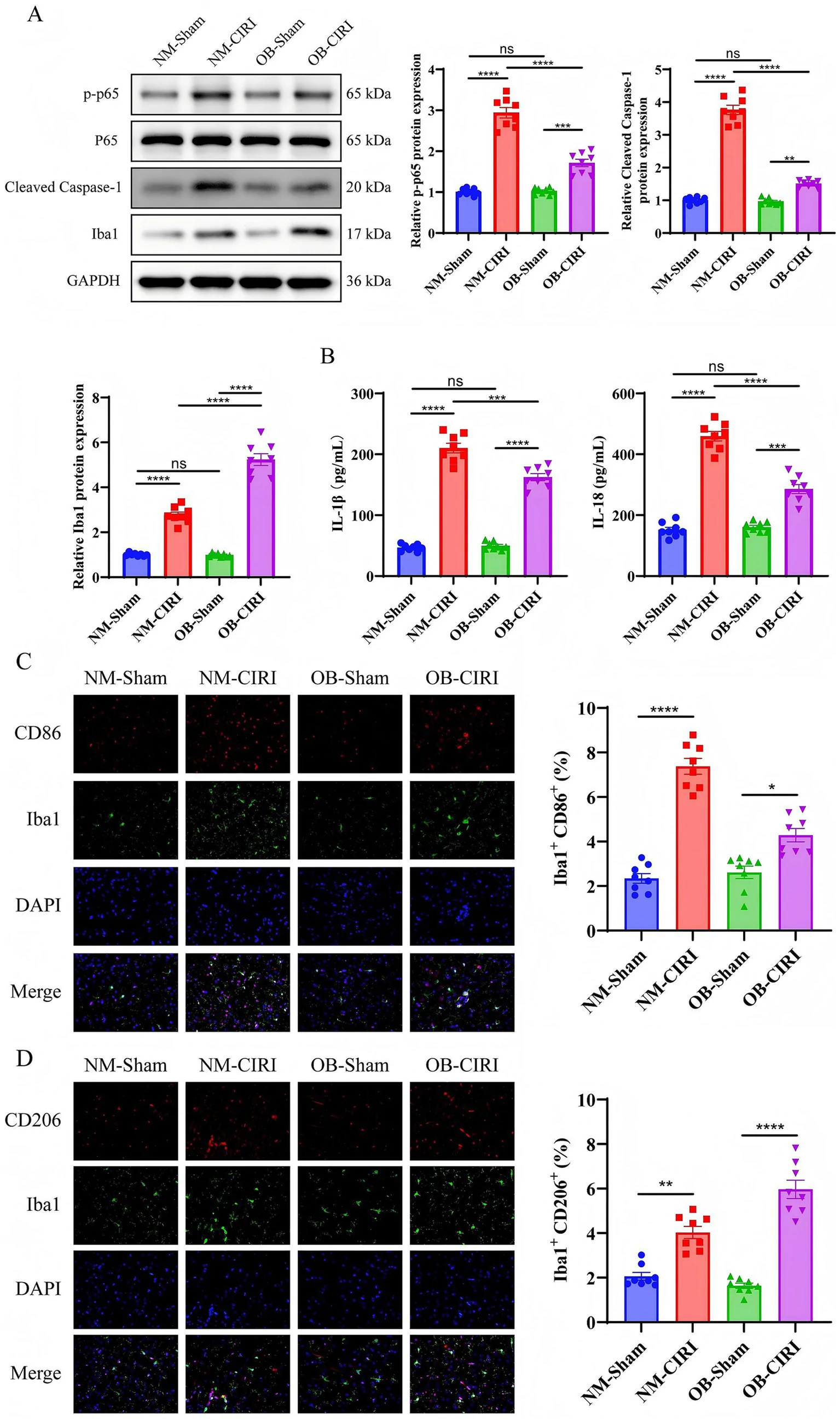
Obesity-associated neuroprotection is linked to inhibition of the NF-κB/NLRP3 pathway and induction of microglial M2 polarization. **(A)** WB analysis and densitification of p-p65, Cleaved Caspase-1, and Iba1 at 72 h after CIRI. **(B)** ELISA results of IL-1*β* and IL-18 at 72 h after CIRI. **(C)** Immunofluorescence staining of Iba1^+^CD86^+^ and Iba1^+^CD206^+^ cells (scale bar = 100 μm). **(D)** Quantification of Iba1^+^CD86^+^/Iba1^+^CD206^+^ ratios. *n* = 8 each group. *p < 0.05, **p < 0.01, ***p < 0.001, ****p < 0.0001.

Compared with the NM-Sham group, protein expression levels of p-p65, Cleaved Caspase-1, and Iba1 were significantly increased in the NM-CIRI group (*p* < 0.0001). Similar increases were observed in the OB-CIRI group compared with OB-Sham (*p* < 0.0001). However, these protein levels were significantly reduced in the OB-CIRI group compared with the NM-CIRI group (*p* < 0.05) (Figure 3A). Levels of IL-1β and IL-18 were significantly elevated in both NM-CIRI and OB-CIRI groups compared with their respective sham groups (*p* < 0.0001), with significantly lower levels in the OB-CIRI group than in the NM-CIRI group (*p* < 0.0001) (Figure 3B). Immunofluorescence analysis showed that Iba1^+^CD86^+^ co-localization was significantly increased in the NM-CIRI group compared with NM-Sham (*p* < 0.05), whereas Iba1^+^CD206^+^ co-localization was markedly increased in the OB-CIRI group compared with OB-Sham (*p* < 0.0001) (Figures 3C,D).

### 3.4 BHB mimics the neuroprotective effects of obesity

**Figure 4:**
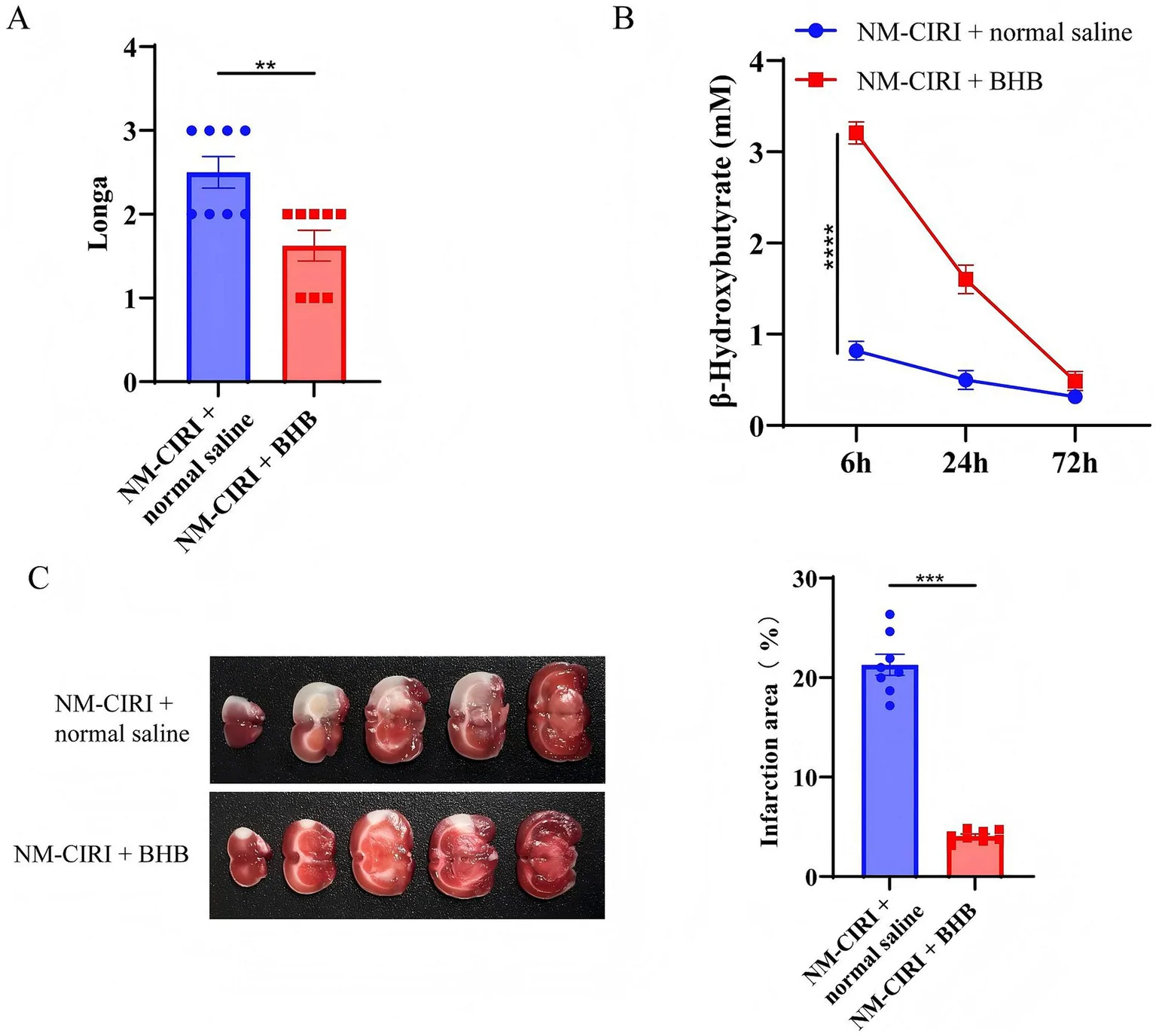
BHB mimics the neuroprotective effects of obesity. **(A)** Zea–Longa scores at 72 h after CIRI. **(B)** Dynamic changes in serum BHB levels at 6 h, 24 h, and 72 h after CIRI. **(C)** TTC staining and infarct volume analysis at 72 h after CIRI. *n* = 8 each group. *p < 0.05, **p < 0.01, ***p < 0.001, ****p < 0.0001. NM-CIRI + normal saline, normal CIRI rats treated with normal saline; NM-CIRI + BHB, normal CIRI rats treated with BHB.

Compared with the NM-CIRI + normal saline group, Zea–Longa scores were significantly reduced in the NM-CIRI + BHB group (*p* < 0.0001) (Figure 4A). At 6 h, 24 h, and 72 h, serum BHB levels in the NM-CIRI + normal saline group gradually declined over time, whereas levels in the NM-CIRI + BHB group remained significantly higher at all time points (*p* < 0.0001), with a slowing rate of increase over time (Figure 4B). Infarct volume was significantly reduced in the NM-CIRI + BHB group compared with the NM-CIRI + normal saline group (*p* < 0.0001) (Figure 4C).

### 3.5 BHB alleviates neuroinflammation by inhibiting NF-κB/NLRP3 activation and promoting microglial M2 polarization

**Figure 5:**
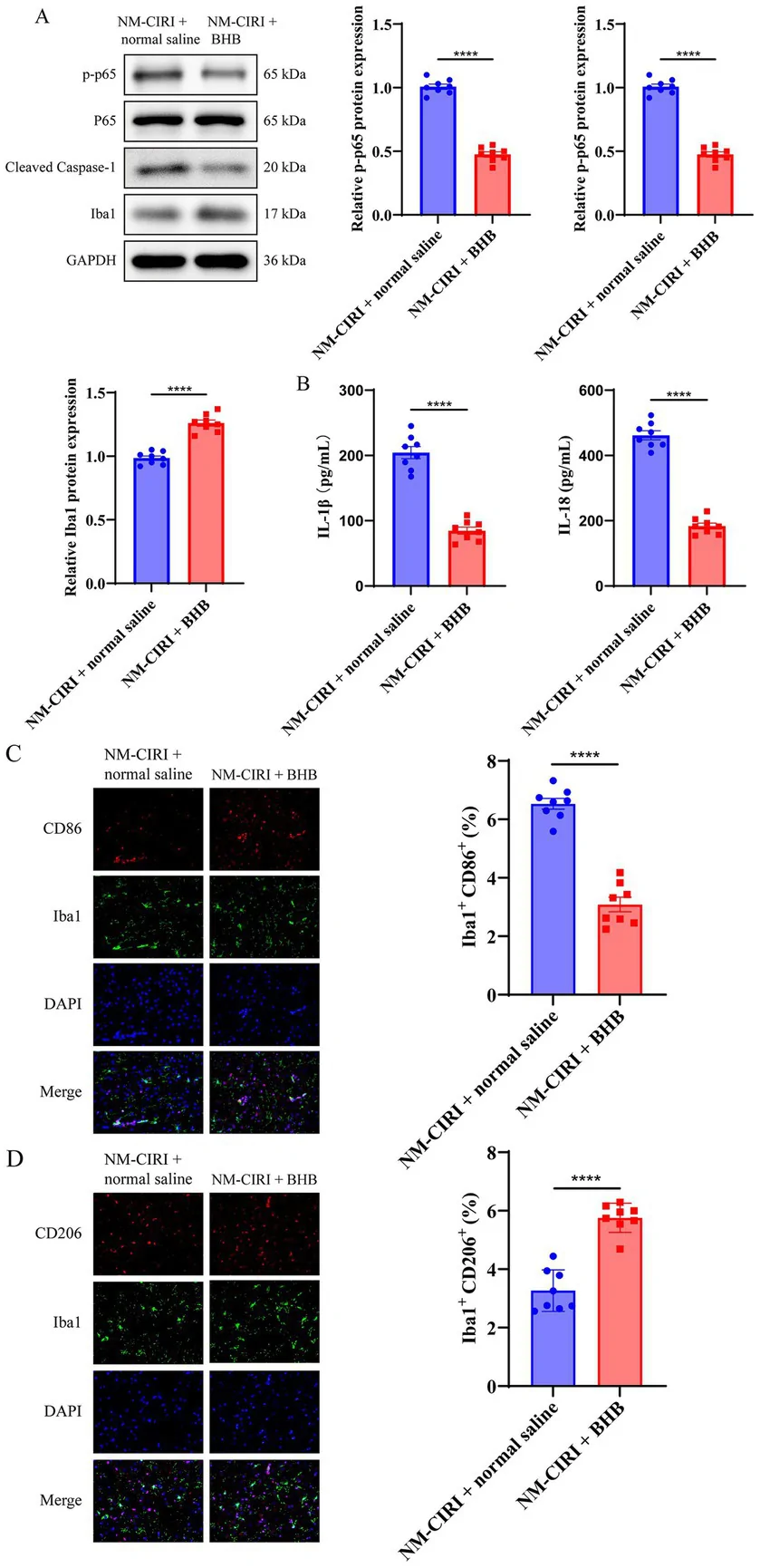
BHB alleviates neuroinflammation by inhibiting NF-κB/NLRP3 activation and promoting microglial M2 polarization. **(A)** WB analysis and densitification of p-p65, Cleaved Caspase-1, and Iba1 at 72 h after CIRI. **(B)** ELISA results of IL-1β and IL-18 at 72 h after CIRI. **(C)** Immunofluorescence staining of Iba1^+^CD86^+^ and Iba1^+^CD206^+^ cells (scale bar = 100 μm). **(D)** Quantification of Iba1^+^CD86^+^/Iba1^+^CD206^+^ ratios. *n* = 8 each group. *p < 0.05, **p < 0.01, ***p < 0.001, ****p < 0.0001.

Compared with the NM-CIRI + normal saline group, the NM-CIRI + BHB group showed significantly decreased expression of p-p65 and Cleaved Caspase-1 (*p* < 0.0001), while Iba1 expression was increased (*p* < 0.05) (Figure 5A). Levels of IL-1β and IL-18 were significantly reduced in the NM-CIRI + BHB group (*p* < 0.0001) (Figure 5B). Immunofluorescence analysis revealed significantly decreased Iba1^+^CD86^+^ co-localization and increased Iba1^+^CD206^+^ co-localization in the NM-CIRI + BHB group (*p* < 0.0001) (Figures 5C,D).

### 3.6 Activation of the NF-κB/NLRP3 pathway reverses BHB-mediated M2 polarization and neuroprotection

**Figure 6:**
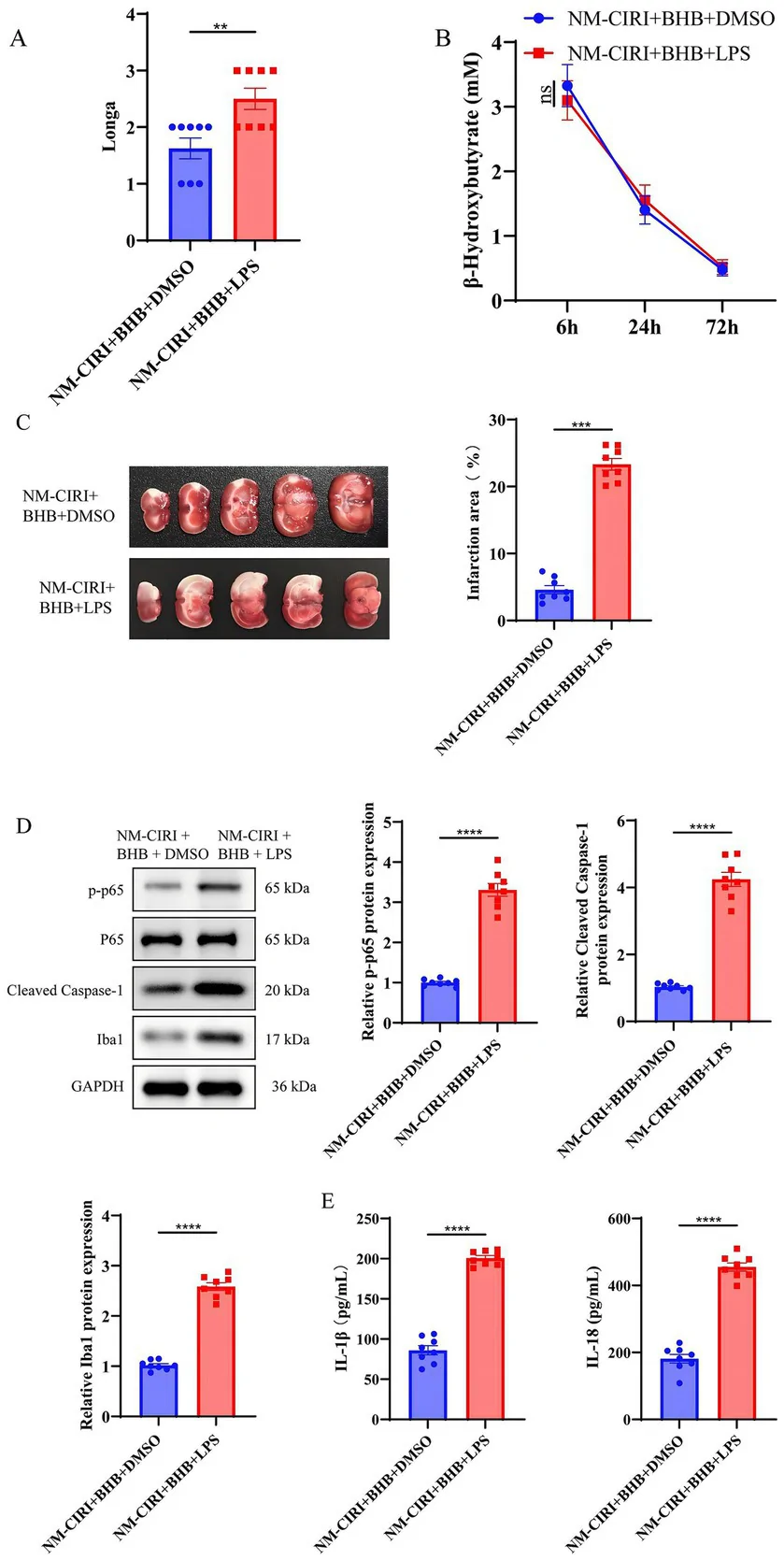
Activation of the NF-κB/NLRP3 pathway reverses BHB-mediated M2 polarization and neuroprotection. **(A)** Zea–Longa scores at 72 h after CIRI. **(B)** Dynamic changes in serum BHB levels at 6 h, 24 h, and 72 h after CIRI. **(C)** TTC staining and infarct volume analysis at 72 h after CIRI. **(D)** WB analysis and densitification of p-p65, Cleaved Caspase-1, and Iba1. **(E)** ELISA results of IL-1β and IL-18 at 72 h after CIRI. *n* = 8 each group. *p < 0.05, **p < 0.01, ***p < 0.001, ****p < 0.0001. NM-CIRI + BHB + DMSO, normal CIRI rats treated with both BHB and DMSO; NM-CIRI + BHB + LPS, normal CIRI rats treated with both BHB and LPS.

Compared with the NM-CIRI + BHB + DMSO group, Zea–Longa scores were significantly increased in the NM-CIRI + BHB + LPS group (*p* < 0.0001) (Figure 6A). Serum BHB levels in the NM-CIRI + BHB + DMSO group decreased over time (*p* < 0.0001), with no significant difference between the LPS and DMSO groups at any time point (Figure 6B). Infarct volume was significantly increased in the NM-CIRI + BHB + LPS group compared with the NM-CIRI + BHB + DMSO group (*p* < 0.0001) (Figure 6C). Moreover, levels of p-p65, Cleaved Caspase-1, and Iba1 were significantly upregulated in the NM-CIRI + BHB + LPS group (*p* < 0.0001) (Figure 6D), accompanied by markedly increased levels of IL-1β and IL-18 (*p* < 0.0001) (Figure 6E).

### 3.7 Activation of the NF-κB/NLRP3 pathway reverses the neuroprotective effect mediated by BHB

**Figure 7:**
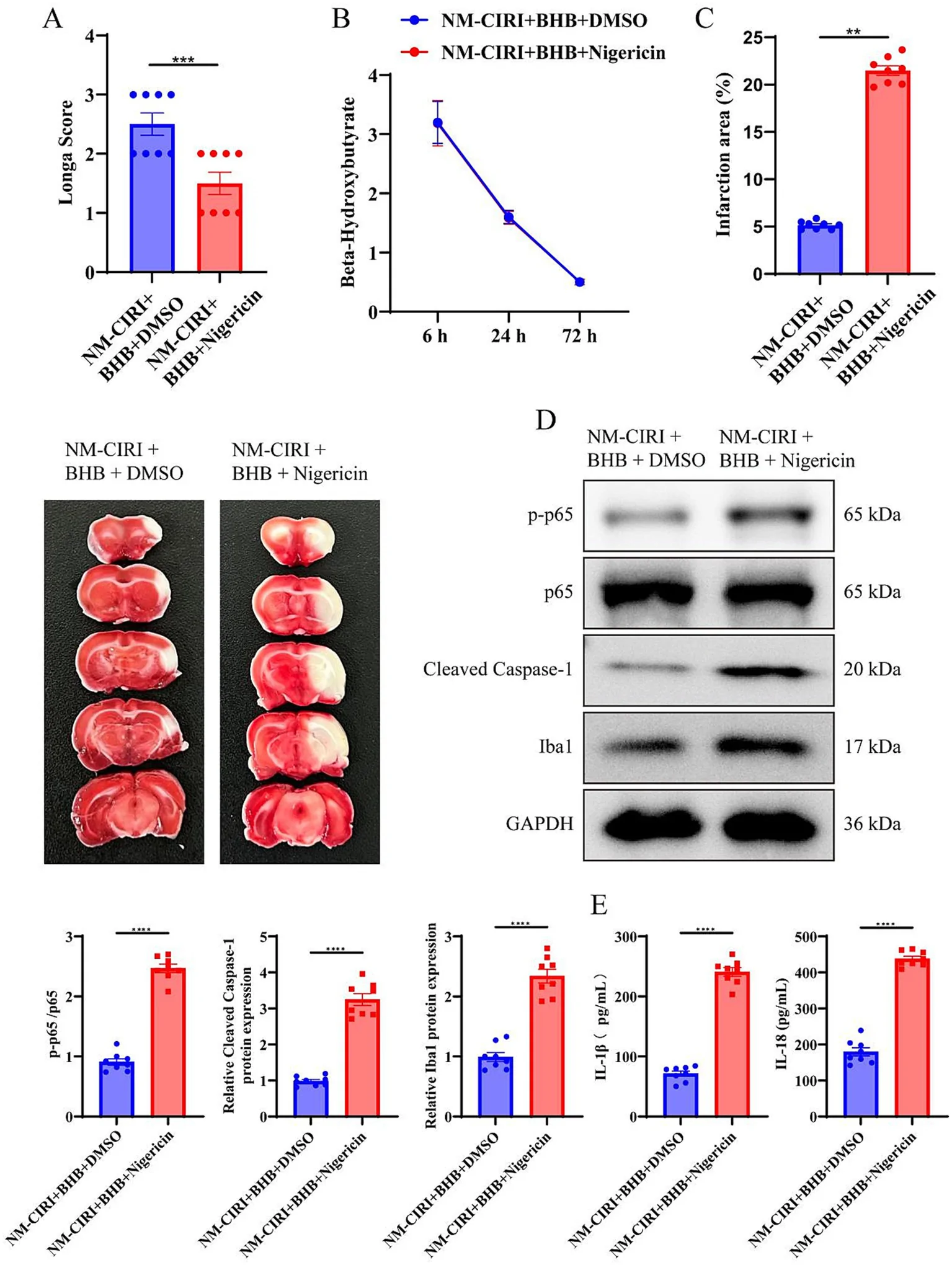
Nigericin abolishes BHB-mediated neuroprotection by activating NLRP3 inflammasome. **(A)** Zea–Longa neurological deficit scores at 72 h after CIRI. **(B)** Dynamic changes of serum β-HB levels at 6 h, 24 h and 72 h after CIRI. **(C)** Quantitative analysis of infarct volume percentage via TTC staining. **(D)** Western blot analysis and quantitative statistics of p-p65, cleaved caspase-1 and Iba1 protein expression. **(E)** ELISA detection of IL-1β and IL-18 levels in brain tissues. *n* = 8 each group. ns, no significance; *p < 0.05, **p < 0.01, ***p < 0.001, ****p < 0.0001. NM-CIRI + BHB + DMSO, normal CIRI rats treated with BHB and DMSO; NM-CIRI + BHB + Nigericin, normal CIRI rats treated with BHB and Nigericin.

Zea–Longa neurological deficit scores were significantly increased in the NM-CIRI + BHB + Nigericin group compared with the NM-CIRI + BHB + DMSO group (*p* < 0.0001), indicating aggravated neurological dysfunction (Figure 7A). Dynamic detection of serum BHB revealed that serum BHB levels in both groups decreased in a time-dependent manner at 6 h, 24 h and 72 h after modeling. No significant differences in BHB concentration were observed between the two groups at all time points (ns) (Figure 7B), suggesting that Nigericin did not affect the metabolic clearance of exogenous BHB. TTC staining of brain tissues showed that the cerebral infarct volume was markedly larger in the NM-CIRI + BHB + Nigericin group than in the NM-CIRI + BHB + DMSO group (*p* < 0.0001) (Figure 7C). Western blot analysis demonstrated that the protein expression levels of p-p65, cleaved caspase-1 and Iba1 were significantly upregulated in the NM-CIRI + BHB + Nigericin group (*p* < 0.0001) (Figure 7D). ELISA results further verified that the secretion levels of pro-inflammatory cytokines IL-1*β* and IL-18 were remarkably elevated in the NM-CIRI + BHB + Nigericin group (*p* < 0.0001) (Figure 7E). Collectively, Nigericin reversed the neuroprotective effects of BHB via excessive activation of the NLRP3 inflammasome.

## 4 Discussion

Cerebral Ischemia–reperfusion Injury (CIRI) is a key pathological process underlying secondary brain injury following recanalization therapy for acute ischemic stroke. Its core mechanisms involve excessive neuroinflammation, imbalance of microglial polarization, and dysregulation of related signaling pathways. Identifying endogenous molecules capable of targeting these processes is a major focus in CIRI research (15). β-hydroxybutyrate (BHB), the principal ketone body *in vivo*, not only serves as an important energy substrate in lipid metabolism but also exhibits independent anti-inflammatory and immunomodulatory properties (16). Under obese conditions, dysregulated lipid metabolism leads to elevated endogenous BHB levels, providing a novel perspective for exploring its role in CIRI. Meanwhile, the NF-κB/NLRP3 pathway is a central regulator of neuroinflammation, closely associated with microglial M1 polarization and pro-inflammatory cytokine release, and represents a classical therapeutic target in CIRI (9, 17). Therefore, this study established obesity and CIRI rat models, combined with exogenous BHB intervention and LPS-induced pathway activation, to investigate the neuroprotective effects and molecular mechanisms of BHB *in vivo*.

The present study demonstrated that neurological deficits and infarct volume were significantly reduced in the OB-CIRI group compared with the NM-CIRI group, accompanied by markedly elevated endogenous BHB levels at 6 h and 24 h. These findings suggest that increased endogenous BHB under obese conditions acts as a compensatory protective factor following CIRI. Although obesity is traditionally considered a risk factor for cerebrovascular diseases, this apparent contradiction reflects the complexity of metabolic regulation and is consistent with the findings of Newman et al. (18). In obesity, enhanced lipid metabolism leads to hepatic acetyl-CoA accumulation and increased ketone body production, with endogenous BHB levels rising by 2–3-fold. The anti-inflammatory properties of ketone bodies may partially counteract obesity-induced vascular endothelial damage. Notably, endogenous BHB elevation was transient, occurring only at 6 h and 24 h after CIRI and returning to baseline by 72 h, which aligns with the pathophysiological progression of CIRI. Previous studies indicate that 0–24 h post-CIRI represents the early inflammatory phase, characterized by rapid microglial activation and cytokine release, followed by gradual resolution after 24 h (19). This suggests that the protective effect of endogenous BHB is time-dependent and primarily acts during the early inflammatory window.

Exogenous BHB administration further confirmed its neuroprotective role. Compared with the normal saline group, BHB significantly reduced neurological deficit scores and infarct volume, while maintaining elevated serum BHB levels at 6 h, 24 h, and 72 h. These findings are consistent with previous reports showing that BHB exerts central neuroprotection by suppressing inflammatory responses and has been shown to reduce ischemic brain injury and improve neurological outcomes in experimental models (20). Moreover, exogenous BHB supplementation reduces infarct volume and improves neurological recovery in mice with acute cerebral infarction (21). Recent evidence further demonstrated that BHB facilitates blood–brain barrier repair after ischemic stroke through epigenetic regulation of tight-junction protein ZO-1 expression, thereby contributing to neurovascular protection and functional recovery (22). Compared with conventional neuroprotective agents for CIRI, such as edaravone and butylphthalide, BHB, as an endogenous metabolite, may offer potential advantages including high biocompatibility, low toxicity, and efficient blood–brain barrier penetration (23). Continuous administration via subcutaneous osmotic pumps ensured stable BHB levels, minimizing fluctuations associated with single-dose delivery and resembling clinical intravenous infusion strategies, thereby providing a reference for future translational applications. Moreover, exogenous BHB prolonged the duration of ketone-mediated protection, compensating for the transient elevation of endogenous BHB.

Mechanistically, BHB significantly reduced the expression of p-p65 and cleaved caspase-1 in brain tissue, inhibited activation of the NF-κB/NLRP3 pathway, and decreased the release of IL-1β and IL-18. Simultaneously, BHB downregulated the M1 marker CD86 and upregulated the M2 marker CD206, promoting microglial polarization toward an anti-inflammatory phenotype. These findings are consistent with previous studies (24) and further clarify the regulatory role of ketone bodies in central neuroinflammatory pathways. BHB acts as an endogenous inhibitor of the NLRP3 inflammasome by directly binding to NLRP3 and suppressing its oligomerization, thereby preventing inflammasome activation and reducing pro-inflammatory cytokine release. The inhibitory effects observed on cleaved caspase-1, IL-1β, and IL-18 support this mechanism. In addition, NF-κB activation is a key driver of M1 polarization, and its inhibition promotes M2 polarization; thus, suppression of p-p65 represents a critical molecular basis for BHB-induced microglial phenotypic shift (25).

To further validate the central role of the NF-κB/NLRP3 pathway, LPS was used to forcibly activate this pathway. LPS administration completely abolished the neuroprotective effects of BHB, including improvements in neurological function and reductions in infarct volume, while restoring NF-κB/NLRP3 activation, increasing pro-inflammatory cytokine levels, and reducing M2 polarization. Notably, LPS did not affect serum BHB levels, indicating that its effects were not mediated by altering BHB concentration but by directly activating the NF-κB/NLRP3 pathway and blocking downstream regulatory effects of BHB. LPS is a classical activator of neuroinflammatory signaling, acting through Toll-like receptor 4 to rapidly activate NF-κB and promote NLRP3 inflammasome activation (26). These findings provide strong evidence that the NF-κB/NLRP3 pathway is a major mechanistic target involved in BHB-mediated neuroprotection in CIRI.

Ionized calcium-binding adapter molecule 1 (Iba1) is a widely used marker of overall microglial activation rather than a specific indicator of polarization phenotype. Increased Iba1 expression reflects activation and proliferation of microglia following cerebral ischemia–reperfusion injury. Therefore, in the present study, Iba1 was used to evaluate the overall activation status of microglia, whereas CD86 and CD206 were employed as complementary markers to characterize polarization toward pro-inflammatory and anti-inflammatory phenotypes, respectively. Our results showed that both obesity-associated endogenous BHB elevation and exogenous BHB administration significantly reduced Iba1 expression, indicating attenuation of excessive microglial activation. Meanwhile, decreased CD86 and increased CD206 expression further suggested a shift toward an anti-inflammatory microglial phenotype.

Several limitations should be acknowledged. First, serum BHB concentrations were determined at 6 h, 24 h and 72 h post-intervention, whereas all molecular detections including western blot, ELISA and immunofluorescence as well as functional evaluations were only performed at the 72 h time point. Such single-time-point molecular measurements fail to clarify the causal link between early metabolic fluctuation and subsequent inflammatory pathway alternations. Without sequential dynamic sampling, it is difficult to distinguish whether NF-κB/NLRP3 pathway inhibition serves as the cause of alleviated cerebral injury or a secondary consequence of improved pathological outcomes. Serial time-gradient detection of pivotal markers is required in future investigations. Second, BHB permeability was not explored in the present work. Additional experiments including Evans blue extravasation assay and tight junction protein expression analysis would further strengthen the mechanistic evidence of BHB-mediated neuroprotection. Third, all experimental conclusions were derived from *in vivo* rat models. Further *in vitro* assays based on cultured microglia or brain endothelial cells are essential to verify the direct regulatory effects of BHB at the cellular level.

In conclusion, BHB exerts neuroprotective effects in CIRI by inhibiting activation of the NF-κB/NLRP3 pathway, reducing IL-1β and IL-18 expression, and promoting microglial polarization toward the anti-inflammatory M2 phenotype, thereby alleviating neurological injury and cerebral infarction.

## Data Availability

The original contributions presented in the study are included in the article/Supplementary material, further inquiries can be directed to the corresponding author.
