# Supplementary material for: β-Hydroxybutyrate induces microglial M2 polarization by inhibiting the NF-κB/NLRP3 pathway to ameliorate cerebral ischemia–reperfusion injury
Source: Front Neurol. 2026 Aug 12;17:1847958. doi: 10.3389/fneur.2026.1847958 (PMC13506300; doi:10.3389/fneur.2026.1847958)
Supplement: Supplementary file 1 [file Image_1.pdf]

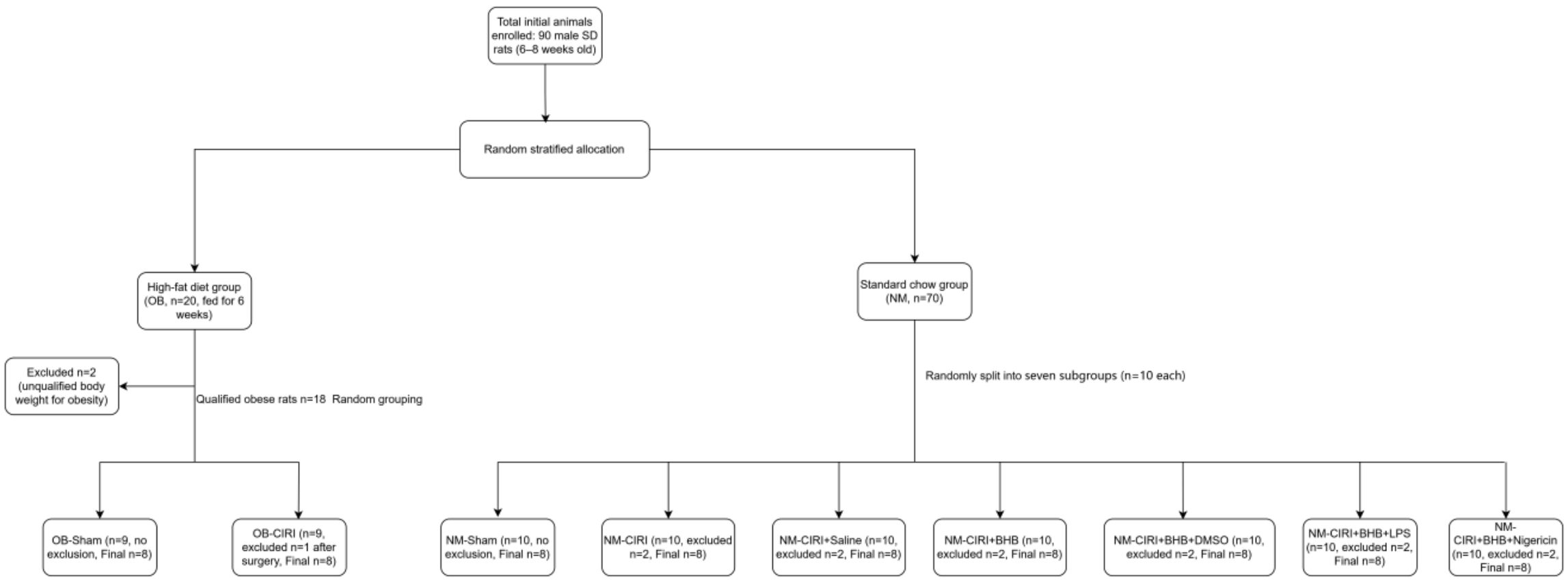

Supplementary Figure S1. CONSORT-style flow chart for animal grouping and exclusion.
